# Corns

**Published:** 1867-12

**Authors:** 


CORNS.

are the botheration of the vast number of silly folk, who for appearance's sake, render themselves liable to purgatoric suffering during the entire space of their natural life thereafter; there are multitudes of cures for corns, the very quickest and most certain method known to us, is to cut off the toe, and be done with it; a better plan, to our own personal knowledge, in reference to all corns, is not to have any; and this exemption can be secured by wearing shoes which can be drawn on the feet without much straining with two pair of woollen stockings, then when you get home pull off one pair and wear the shoe. But, as some poet has written, that variety is the spice of life, various "infallible" cures for corns are appended for the amusement and practice of our afflicted reader.

"Pare the corn as close as you can, then get a thin piece of India rubber cloth, about the twentieth of an inch thick, (the pure India rubber is the best, but that made of cotton will do) and where the corn is on one of the toes, make a stall of it; or where it is on another part of the foot, sew it on the inside of the stocking and large enough to cover the corn well. By continuing the application from four to six weeks, and paring the corn as the callous skin loosens, the corn will disappear. The application of the rubber will give im mediate relief to the pain. The principle ef the cure is to assist nature in restoring the skin to its natural condition again."

One objection to the above is, it is too tedious and troublesome; another more serious is that a number of cases are recorded in medical works where the paring of a corn has been followed by a fatal bleeding or inflammation, irritation, lockjaw and an agonizing death.

—Scrape a piece of common chalk, put a small portion of it upon the corn and bind it with a linen rag. Repeat the application for a few days, and you will find the corn come off like a shell, and perfectly cured. The cure is simple and efficious.

—Mr. Wakely, in the Royal Free Hospital, London, is in the habit of applying glycerine to corns. It softens those excrescences so that they may be scooped out with ease.

—Take a piece of lemon, nick it so as to let in the toe with the corn, tie this at night so that it cannot move, and you will find the next morning that, with a blunt knife, the corn will come away to a great extent.

